# Is Urinary Lipoarabinomannan the Result of Renal Tuberculosis? Assessment of the Renal Histology in an Autopsy Cohort of Ugandan HIV-Infected Adults

**DOI:** 10.1371/journal.pone.0123323

**Published:** 2015-04-21

**Authors:** Janneke A. Cox, Robert L. Lukande, Sam Kalungi, Eric Van Marck, Koen Van de Vijver, Andrew Kambugu, Ann M. Nelson, Robert Colebunders, Yukari C. Manabe

**Affiliations:** 1 Department of Clinical Sciences, Institute of Tropical Medicine, Antwerp, Belgium; 2 Infectious Diseases Institute, Makerere University College of Health Sciences, Kampala, Uganda; 3 Department of Pathology, College of Health Sciences, Makerere University, Kampala, Uganda; 4 Department of Pathology, Mulago Hospital Complex, Kampala, Uganda; 5 Department of Pathology, University Hospital Antwerp, University of Antwerp, Belgium; 6 Department of Diagnostic Oncology & Molecular Pathology, Netherlands Cancer Institute—Antoni van Leeuwenhoek Hospital, Amsterdam, The Netherlands; 7 Joint Pathology Center, Silver Spring, United States of America; 8 Department of Epidemiology and Social Medicine, University of Antwerp, Belgium; 9 Division of Infectious Diseases, Department of Medicine, Johns Hopkins University School of Medicine, Baltimore, Maryland, United States of America; Emory University, UNITED STATES

## Abstract

**Objective:**

The detection of urinary lipoarabinomannan (LAM), a mycobacterial cell wall component, is used to diagnose tuberculosis (TB). How LAM enters the urine is not known. To investigate if urinary LAM-positivity is the result of renal TB infection we correlated the outcomes of urinary LAM-antigen testing to renal histology in an autopsy cohort of hospitalized, Ugandan, HIV-infected adults.

**Methods:**

We performed a complete autopsy, including renal sampling, in HIV-infected adults that died during hospitalization after written informed consent was obtained from the next of kin. Urine was collected postmortem through post-mortem catheterisation or by bladder puncture and tested for LAM with both a lateral flow assay (LFA) and an ELISA assay. Two pathologists assessed the kidney histology. We correlated the LAM-assay results and the histology findings.

**Results:**

Of the 13/36 (36%) patients with a positive urinary LAM ELISA and/or LFA, 8/13 (62%) had renal TB. The remaining 5 LAM-positive patients had disseminated TB without renal involvement. Of the 23 LAM-negative patients, 3 had disseminated TB without renal involvement. The remaining LAM-negative patients had no TB infection and died mostly of fungal and bacterial infections. LAM LFA had a sensitivity of 81% and specificity of 100% to diagnose TB at any location, and the LAM ELISA a sensitivity of 63% and a specificity of 100%. 54% (7/13) LAM LFA-positive patients were not on anti-TB treatment at the time of death.

**Conclusion:**

Renal TB infection explained LAM-positivity in the majority of patients. Patients with disseminated TB without renal involvement can also be diagnosed with LAM. This suggests that other mechanisms that lead to urinary LAM-positivity exist in a minority of patients.

## Introduction

In 2012, the estimated number of TB-HIV co-infected patients in sub-Saharan Africa (SSA) was 825,000 and 320,000 co-infected patients died. Uganda has a high HIV and TB burden, and in 2013 an estimated 48% of TB patients were co-infected with HIV [1–3].

Diagnosing TB in HIV co-infected patients is challenging [4]. Methods relying on mycobacterial detection all have important flaws. Sputum microscopy remains the most commonly used method in SSA, however its sensitivity is low [5, 6]. Culture is the gold standard, but is not widely available and has a relatively long turn-over time [7]. Mycobacterial DNA detection has higher diagnostic sensitivity and provides results the same day, but in resource poor settings, limited availability, technical requirements and costs are important disadvantages [8–10]. Another diagnostic approach is antigen detection: lipoarabinomannan (LAM), a lipopolysaccharide component of the mycobacterial cell wall, can be detected in the urine of TB-infected patients [11–15]. Overall, diagnostic sensitivity of the ELISA LAM assay in urine is poor. But in HIV-infected patients with low CD4 cell counts (<50 cells/μL) sensitivity increases to 56–85% [11, 12, 16–18]. Recently a point-of-care test for LAM in urine is developed, the Determine TB LAM lateral flow assay (LFA) (Alere, Waltham, MA, USA). This immunochromatographic assay attaches colloidal gold-labelled antibodies to LAM that are captured by immobilized LAM antibodies further along the test strip and form a visual band. LFA diagnostic accuracy is comparable to the ELISA assay in HIV-TB co-infected individuals and increases when CD4 cell counts decrease [18–20].

There is an incomplete understanding of how LAM enters the urine. A South African study detected mycobacterial DNA in half of the LAM-positive urinary samples of HIV-infected patients, suggesting that LAM-antigenuria represents whole mycobacteria in the urine[21]. African autopsy series found renal TB in 39–52% of TB-HIV co-infected adults not on antiretroviral treatment [22–24]. Since extra-pulmonary and disseminated TB are more frequent in severely immunosuppressed HIV-infected patients, renal TB could explain the higher sensitivity of urinary LAM assays in this population.

We sought to investigate if urinary LAM-positivity is the result of renal TB infection. We correlated the outcomes of urinary LAM-antigen testing to the histological findings in renal tissue in an autopsy cohort of hospitalized, Ugandan, HIV-infected adults.

## Methods

### Ethics Statement

The study received ethical approval from the Joint Clinical Research Center Research and Ethics Committee (Uganda), the Mulago Internal Review Board (Uganda) and the Institute of Tropical Medicine Institutional Review Board (Belgium). The study was registered by the Uganda National Council of Science and Technology (HS1300).

### Setting and population

This study is a sub-study of a larger autopsy study that was conducted from February—June 2013 in Mulago hospital, a tertiary-care hospital in Kampala. The methods used are described in detail elsewhere[25]. In brief, HIV-infected adults (>18 years old) that died on one of the medicine wards were included after written informed consent was obtained from their next of kin. Post-partum deaths and deaths after trauma were excluded.

### Autopsy and histological assessment

A complete autopsy took place within 4 hours after consent was obtained. Urine was collected in a sterile container through post-mortem catheterisation or by puncturing the bladder during the autopsy and was stored at -20°C directly after the procedure. The autopsy included routine sampling of both kidneys (samples containing cortex, medulla and corticomedullary junction) and additional sampling in case of any macroscopically abnormal lesion. Tissue was fixed in 10% formalin.

Hematotoxylin and eosin (H&E) slides were made for each tissue section and read by 2 experienced pathologists. In case of discrepancy, discussion between the two pathologists would provide consensus. The general kidney histology was assessed on H&E stained slides. Additional stains, including Ziehl-Neelsen (ZN), Periodic Acid-Schiff diastase and Jones silver staining were done when requested by the pathologists. For the final (renal) diagnoses, the histological findings were combined with the available clinical and macroscopic data.

TB was defined as the presence of acid-fast bacilli (AFB) in any organ or granuloma formation with presence of giant cells or (caseous) necrosis not otherwise explained. Renal TB was defined as the presence of AFB in the kidneys or granuloma formation with presence of giant cells or (caseous) necrosis not otherwise explained. HIV-associated nephropathy (HIVAN) was defined as a constellation of glomerular, interstitial and tubular abnormalities or the presence of microcysts. If only tubular or interstitial disease was present, additional epithelial cell hyperplasia or hypertrophy was required. Acute tubular necrosis (ATN) was defined as massive coagulation necrosis of tubular epithelial cells and presence of granular casts.

The pathologists reading the slides were blinded to the outcome of the LAM-antigen testing.

### LAM testing

After all autopsies were performed, the urine samples were thawed to ambient temperature. One millilitre of urine was heated to 95–100°C for 30 minutes and when cooled down, spun 15 minutes (10,000 rpm.). The supernatant was collected. LAM-antigen testing was done on an unprepared defrosted sample, further referred to in the text as unprepared urine, and on the supernatant. For the LFA testing, the Determine TB LAM was used and for ELISA testing, the Clearview TB ELISA. Both tests were provided by Alere (Waltham, MA, USA).

For each collected urine sample, 60μL of unprepared urine and 60μL of supernatant was applied to a LFA test strip. After 25 minutes, 2 experienced lab technicians read the test strip by comparing the LFA test result to the manufacturer-supplied reference card (grading from +1 to +5). The +1 cut-off point was used for positivity. In case of discrepancy between the two readers, immediate discussion between them provided consensus.

For the ELISA assay, 0.1ml of supernatant was applied in duplicate on a 96-well plate. Testing procedures were conducted according to manufacturer’s instructions, which included a duplicate positive and negative control on each plate. Immediately after processing, optical density (OD) was measured at 450nm. The average OD for each sample was compared to the cut-off for positivity/negativity. The cut-off was determined by adding 0.1 OD units to the average negative control OD value, as instructed by the manufacturer.

The lab technicians were blinded to the autopsy results.

### Statistical methods

Data was analyzed using STATA version 11.0 (Stata Corp., Texas, USA). Proportions are reported with 95% confidence intervals (CI) and non-normal distributed continuous variables as a median with interquartile ranges (IQR). When comparing proportions, a Chi square or Fisher’s exact test was performed and when comparing non-parametric numerical variables, a Mann-Whitney test. A p-value <0.05 was considered statistically significant.

## Results

### Patient characteristics

We collected urine from 38 autopsy-cases. One patient was excluded from further analysis because only one kidney block was retrieved after tissue processing. Another patient was excluded because no differentiation could be made between disseminated (treated) *Cryptococcus neoformans* infection and TB. This patient died 10 days after completing a 2-week course of amphotericin-B and fluconazole for culture positive cryptococcal meningitis and had macroscopically nodular growths in multiple organs and microscopically necrotic granulomas in the lungs, the spleen and lymph nodes but additional staining with Grocott silver and ZN did not reveal any causative microorganism.

The median age was 38 years (IQR 32–43), the median CD4 count 39 cells/μL (IQR 13–64). Twenty patients (56%) were reported to be on antiretroviral therapy (ART) for a median duration of 21 days (IQR 14–183). Eight cases (22%) were reported to be on anti-TB treatment for a median duration of 14 days (IQR 5–42). Characteristics were similar in TB-infected LAM-positive, TB-infected LAM-negative and TB-uninfected patients, although a trend of higher CD4 cell counts in TB-infected LAM-negative patients was observed when compared to TB-infected LAM-positive patients (p = 0.07) (Table 1). Only 46% of the LAM-positive patients were on TB-treatment.

**Table 1:** Patient characteristics.

	Total (n = 36)	TB-infected (n = 16)		TB-uninfected* (n = 20)
		LAM-positive (n = 13)	LAM-negative (n = 3)	
Gender (% female)	42	31	33	50
Median age (yrs; IQR)	38 (32–43)	39 (30–47)	38 (range 33–39)	37 (33–42)
Median CD4 count (cells/μL; IQR)	39 (13–64)	19 (11–39)**	63 (range 47–101)**	41 (17–205)
On ART (%)	56	62	0	60
Median duration of ART (days; IQR)	21 (14–183)	21 (14–21)	-	148 (14–1095)
On anti-TB treatment (%)	22	46	0	10
Median duration anti-TB treatment (days; IQR)	14 (5–42)	8 (5–42)	-	52 (14–90)
Median duration admission (days; IQR)	6 (2–11)	6 (2–9)	11 (range 3–11)	5 (2–11)

n: absolute number; yrs: years; IQR: inter-quartile range; ART: anti retroviral therapy; TB: tuberculosis

* including one patient with granulomatous infection not specified

**p = 0.07.

### Urinary LAM and kidney histology

**Fig 1:**
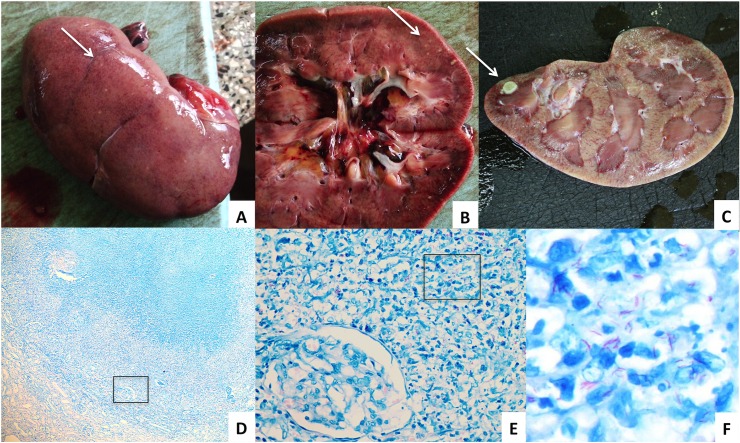
Macro-and microscopic images of TB in the kidney. A. Nodules on outer surface of the right kidney and B. in the kidney parenchyma C. Tuberculoma in the kidney parenchyma D. Ziehl-Neelsen stain of a granuloma in the renal parenchyma showing glomeruli and tubules (x5) E. Microscopic zoom of indicated area with a glomerulus and multiple acid fast bacilli (x40) F. Microscopic zoom of indicated area with multiple acid fast bacilli (x100).

Thirteen patients had a positive LAM ELISA and/or LFA. Of these, 8 (62%) had histological abnormalities compatible with renal TB: 6 with AFB and 2 without AFB on ZN staining of the renal tissue (Table 2 and Fig 1). The remaining 5 LAM-positive patients had disseminated TB, but no histological abnormalities suggestive of renal TB: one had macroscopic white nodules on one kidney but no microscopic lesions suggesting an infection and one had lymphocytic infiltrates on renal histology, a non-specific finding, and additional ZN staining was negative for AFB.

**Table 2:** Characteristics, laboratory outcomes and histological findings per case.

CHARACTERISTICS				LAB TESTING			HISTOLOGY		
				LFA/ intensity		ELISA**	KIDNEY		AUTOPSY DIAGNOSIS
Age Sex	CD4	On ART/ duration*	On TB-tx/ duration*	Unpre-pared	Super- natant		TB-specific abnormalities	Other abnormalities	
**ELISA POSITIVE AND LFA POSITIVE**									
47 M	-	No	No	P / 5	P / 5	P/1.911	Large granuloma, AFB+	Moderate-severe atherosclerosis	Disseminated TB, AFB+ kidney
30 M	12	Y / 21	No	P / -	-	P/2.328	Large granuloma, AFB+	None	Disseminated TB, AFB+ liver and lymphnode
39 M	-	Y / 7	No	P / 4	P / 4	P/0.777	Granuloma, AFB+	None	Disseminated TB, AFB+ spleen
39 M	-	Y / -	No	P / 5	P / 4	P/2.723	Granuloma in cortex, AFB+	Protein casts in tubuli, no glomerulo- or tubulopathy	Disseminated TB, AFB+ lung and brain
30 F	-	No	No	P / 5	P / 5	P/2.26	Multiple granulomas, AFB+	Protein casts in tubuli, no glomerulo- or tubulopathy	Disseminated TB, AFB+ kidney
40 M	24	Y / 21	Y / 42	P / 5	P / 5	P/1.707	Multiple granulomas, AFB-	Protein casts in tubuli, no glomerulo- or tubulopathy	Disseminated TB, AFB+ lung
48 M	64	No	Y / -	P / -	P / 5	P/2.71	One small granuloma, AFB-	Moderate—severe atherosclerosis	Disseminated TB AFB+ lymph node Intracerebral hemorrhage
28 M	-	Y / 196	Y / 5	P / 5	P / 5	P/2.527	None	Protein casts in tubuli, dilated tubuli, no glomerulopathy	Disseminated TB, AFB+ brain and spleen Disseminated CN infection
59 F	29	No	Y / 2	P / 5	P / 5	P/3.645	None	Aspecific lymphocytic infiltrates Moderate atherosclerosis, ATN	Disseminated TB, AFB+ lung
25 M	48	Y / -	Y / 8	P / 4	P / 4	P/0.877	None	HIVAN with severe glomerulopathy	Disseminated TB, AFB-
**LFA POSITIVE AND ELISA NEGATIVE**									
32 M	13	Y / 14	No	P / 4	P / 3	N/0.136	Multiple granulomas, AFB+	None	Disseminated TB, AFB+ lung and liver
48 F	4	No	No	P / 1	P / 2	N/0.201	None	Protein casts in tubuli, no glomerulo- or tubulopathy	Disseminated TB, AFB+ lymphnode Disseminated CN
29 F	9	Y / 28	Y / 42	P / 1	P / 2	N/0.165	None	HIVAN with minor glomerulopathy and ATN	Severe steatohepatitis with liver failure Disseminated TB, AFB-
**ELISA NEGATIVE AND LFA NEGATIVE**									
33 M	47	No	No	N	N	N/0.183	None	HIVAN with moderate-severe glomerulopathy, chronic pyelonefritis and atherosclerosis	Disseminated Kaposi’s sarcoma Disseminated TB, AFB+ lung
39 M	63	No	No	N	N	N/0.189	None	None	Disseminated TB AFB+ meninges
38 F	101	No	No	N	N	N/0.218	None	ATN with hydronephrosis	Disseminated TB, AFB+ spleen
31 M	-	No	No	N	N	N/0.175	None	None	*Pneumocystis jerovecii* pneumonia
32 M	17	Y / 112	No	N	N	N/0.178	None	Some autolysis	Bacterial meningitis and pneumonia Disseminated CN
24 F	-	No	No	N	N	N/0.154	None	None	Liver failure probably alcoholic Intraventricular hemorrhage
39 F	42	Y -	No	N	N	N/0.175	None	None	Liver cirrhosis etiology unknown
30 F	7	Y / 1095	Y / 90	N	N	N/0.157	None	Multiple small cysts	Liver failure etiology unknown
35 M	-	Y -	No	N	N	N/0.202	None	Atherosclerosis and old renal infarction, dilated lymph vessels	Disseminated Kaposi’s sarcoma
62 M	-	Y /-	No	N	N	N/0.142	None	Atherosclerosis and recent renal infarction	Myocardial infarction
45 M	201	Y / -	No	N	N	N/0.207	None	None	Sarcoma
37 M	-	No	No	N	N	N/0.195	None	CN	Disseminated CN
39 F	-	Y / -	No	N	N	N/0.221	None	CN	Disseminated CN
26 F	160	No	-	N	N	N/0.184	None	None	Candida meningitis
38 M	-	Y / 183	No	N	N	N/0.134	None	None	Disseminated Kaposi’s sarcoma
24 F	205	No	No	N	N	N/0.172	None	None	Bacterial sepsis
35 F	-	Y / 3	Y / 14	N	N	N/0.186	None	ATN	Bilateral *Aspergillus* pneumonia
39 F	341	Y / 14	No	N	N	N/0.201	None	None	Massive steatohepatitis
35 F	2	No	No	N	N	N/0.173	None	CN	Disseminated Kaposi’s sarcoma Disseminated CN
63 M	39	No	No	N	N	N/0.162	None	None	Chronic meningitis of unknown etiology Bacterial pneumonia
37 F	638	Y / 2190	No	N	N	N/0.196	None	Atherosclerosis and multiple recent infarctions, no glomerulosclerosis	Aspiration pneumonia
61 M	-	Y / 2	No ***	N	N	N/0.187	None	Atherosclerosis, possibly early HIVAN	Bacterial pneumonia
45 F	-	No	No	N	N	N/0.205	None	Chronic pyelonephritis, atherosclerosis	Bacterial pneumonia

* in days ** Average optical density, cut-off point for positivity was 0.291 *** recently completed 6 months anti-TB treatment; LFA: lateral flow assay; ELISA: enzyme-linked immunosorbent assay; ART: antiretroviral therapy; TB: tuberculosis; M: male; F: female;-: exact intensity unknown but ≥1; Y: yes; P: positive; N: negative; AFB: acid fast bacilli, CN: *Cryptococcus neoformans* infection; HIVAN: HIV-related nephropathy; ATN: acute tubular necrosis.

The general kidney histology of the LAM-positive patients with renal TB showed normal histology in 3/8, protein casts without tubulo- or glomerulopathy in 3/8 and atherosclerosis in 2/8. The kidney histology of the LAM-positive patients without renal TB showed minor-severe glomerulopathy as part of HIVAN in 2/5, ATN and atherosclerosis in 1/5, protein casts without tubulo- or glomerulopathy in 1/5 and protein casts with dilated tubules in 1/5 (Table 3).

**Table 3:** Renal histology in TB-infected patients according to LAM-assay outcome.

	Renal TB	Other renal abnormalities
**LAM+n = 13**	Yes n = 8	None n = 3
		Protein casts in tubuli without glomerulo- or tubulopathy n = 3
		Moderate-severe atherosclerosis n = 2
	No n = 5	HIVAN with minor-severe glomerulopathy n = 2
		Aspecific lymphocytic infiltrates, ATN, moderate atherosclerosis n = 1
		Protein casts in tubuli, dilated tubuli, no glomerulopathy n = 1
		Protein casts in tubuli without glomerulo- or tubulopathy n = 1
**LAM-n = 3**	No n = 3	HIVAN, chronic pyelonephritis, atherosclerosis n = 1
		ATN n = 1
		None n = 1

n: absolute number; +: positive;-: negative; HIVAN: HIV-associated nefropathy; ATN: acute tubular necrosis.

Twenty-three patients (64%) had negative results in both LAM assays. Of these, 3 patients had disseminated TB without histological abnormalities compatible with renal TB. None of these 3 patients was on anti-TB treatment. Their general kidney histology showed moderate-severe glomerulopathy as part of HIVAN, chronic pyelonephritis and atherosclerosis (n = 1), ATN (n = 1), and no abnormalities (n = 1) (Tables 2 and 3).

The remaining 20 LAM-negative patients had no TB infection. The cause of death in these patients included disseminated fungal infections (*Cryptococcus neoformans*, *Aspergillus* and *Candida*), bacterial infections and malignancies (Kaposi’s sarcoma).

### LAM lateral flow assay and LAM ELISA outcomes

In 10 patients, both LAM-assays were positive (Table 2). In these 10, LFA intensities ranged from +4 to +5 and ELISA optical densities ranged from 0.777–3.645 (cut-off point for positivity OD 0.291). Another 3 patients had a positive LFA and a negative ELISA. Of these, LFA intensities ranged from +1 to +4 and ELISA OD from 0.136–0.201. No difference was found in LFA outcome between the unprepared and the supernatant urine samples; a 1-grade decrease of intensity was noted in 2 cases and a 1-grade increase in 2 cases. For the ELISA and LFA negative cases the ELISA OD ranged from 0.134–0.221.

### Urinary LAM results in TB-infected patients

Of the 36 patients, 16 (44%) died of disseminated TB, all but 2 with AFB on ZN staining (Table 2). LAM LFA had a sensitivity of 81% (13/16) and specificity of 100% (20/20) to diagnose TB at any location, and the LAM ELISA a sensitivity of 63% (10/16) and a specificity of 100% (20/20). Fifty-four percent of the LAM-positive patients were not receiving TB-treatment.

## Discussion

The majority of patients with positive urinary LAM results had histological evidence of renal TB infection. In these patients, urinary LAM-positivity is probably the result of renal TB infection and reflects the presence of whole mycobacteria.

**Fig 2:**
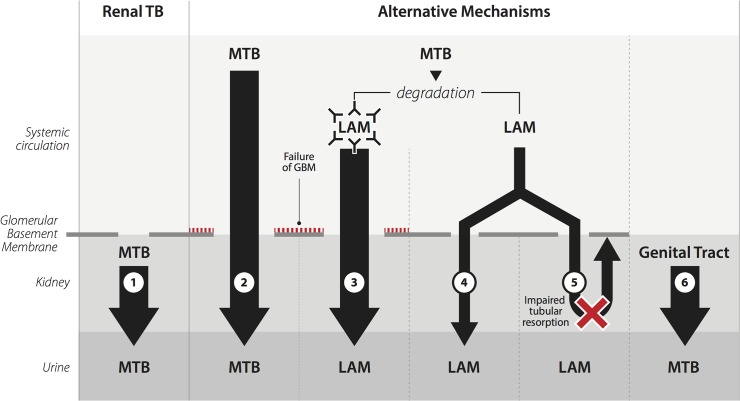
Mechanisms leading to lipoarabinomannan antigenuria. 1. Renal TB 2. Passage of whole *Mycobacterium tuberculosis* through the glomerular basement membrane into the urine 3. Passage of LAM captured by immune-complexes through the glomerular basement membrane into the urine 4. Passage of uncomplexed LAM through the glomerular basement membrane into the urine 5. Impaired tubular resorption of uncomplexed LAM 6. Genital tract TB (Fig. based on [21]). MTB: *Mycobacterium tuberculosis*; GBM: glomerular basement membrane; LAM: lipoarabinomannan.

However, 5 LAM-positive cases (38%) had disseminated TB without evidence of renal TB lesions. In theory, renal TB infection could have been present in these patient but remained undiagnosed because of sampling error (due to inadequate sampling of the lesion, loss of tissue during processing and/or too superficial cutting of the tissue) or atypical histological presentation. However, attention was paid to adequate sampling and tissue processing. Therefore, other mechanisms that led to LAM-antigenuria may have been involved in these patients (Fig 2).

Direct entry from the bloodstream into the urine of *Mycobacterium tuberculosis* (MTB) seems unlikely, due to its size compared to the size of the filtration slits of an intact glomerular basement membrane (GBM) (Fig 2, Mechanism 2)[26]. LAM is immunogenic and anti-LAM antibodies were detected in both urinary LAM-positive and negative HIV-TB co-infected patients[27, 28]. LAM caught in immune-complexes would also be too large the pass an intact GBM (Fig 2, Mechanism 3). Glomerulopathy leading to GBM leakage and proteinuria would allow presence of large molecules in urine. However, we found glomerulopathy in only 2 of the 5 LAM-positive cases without renal TB and in 1 of the 3 LAM-negative TB-infected cases. Moreover, studies that assessed proteinuria found a weak (odds ratio 1.63 (95%CI 1.06–2.52)) or no correlation with LAM-positivity[11, 19, 21, 29].

LAM unbound to immune-complexes can readily pass the GBM (Fig 2, Mechanism 4 and 5)[30]. Uncomplexed LAM could circulate as a result of decreased ability to mount an immunoglobulin response or as the result of a misbalance in LAM-quantity compared to immunoglobulin. If impaired tubular resorption plays any additional role is unknown (Fig 2, Mechanism 5). Lastly, urogenital TB infection beyond the kidney parenchyma could lead to LAM-antigenuria, however isolated TB infection of the ureter and bladder without renal involvement seems non-existent and isolated TB infection of the male genital tract has been described only sporadically (Fig 2, Mechanism 6) [31, 32]. We routinely inspected ureter, urinary bladder and prostate and sampled them in case of macroscopic abnormality. As no abnormalities suggestive of TB were observed, this mechanism seems unlikely.

Mycobacterial load is an important predictor of LAM-antigenuria and provides additional support that either renal TB and/or entry of uncomplexed LAM is the cause of LAM-antigenuria. In addition, anti-TB treatment negatively affects urinary LAM detection [12, 21, 33]. More than one mechanism may be involved in LAM antigenuria and different mechanisms can co-exist within one patient. To further differentiate between the mechanisms, urine of LAM-positive patients should be examined to see if whole mycobacteria, individual LAM polysaccharides or both are excreted. Also 24-hour urinary protein excretion should be measured.

One criticism of the LAM lateral flow assay has been the decreased specificity compared to sputum and blood culture gold standards. In our study, the LAM LFA was 100% specific. Cross-reactivity of the LAM test has been described with urogenital *Candida sp*. [11]. Also dust, soil and feces increased optical density in LAM ELISA, leading to false positivity of the assay [34]. The absence of false-positive results in our study might be the result of the methods we used to obtain the urine (postmortem catheter or bladder puncture) leading to relatively uncontaminated urine samples.

The LAM LFA had a sensitivity of 81% to diagnose TB compared to the histologic gold standard and was more sensitive than the ELISA test. This sensitivity is higher than reported in clinical studies, probably because of our population of terminally ill, severely immune-suppressed patients[18–20].

No difference was found in LFA outcome when using unprepared or supernatant urine. Therefore, heating and spinning of the urine with the objective to separate antigen-antibody complexes did not improve diagnostic accuracy of LFA in our samples.

Lastly, we noted that less than half of the LAM LFA positive patients in our study were on anti-TB treatment. The clinical use of LAM LFA in these patients may have allowed for a timelier diagnosis and prompt treatment.

There are several limitations to our study. We used, as most other studies, defrosted urine samples to allow batch testing. Whether this negatively influenced LAM test results is unknown. Moreover, we were unable to perform culture and/or PCR on our urine samples. This would have allowed us to confirm the presence of whole mycobacteria in the urine and to differentiate between MTB and non-tuberculous mycobacteria (NTM). ELISA LAM positivity in urine is described in cases of *Mycobacterium bovis* infection. For other NTM infections, sensitivity was 5–1000 fold lower compared to MTB[35]. To assess renal histology, we used conventional microscopy and no electron microscopy. Subtle glomerular abnormalities and minimal change nephropathy could therefore been missed. Lastly, we relied on the inpatient ward charts for clinical information. Often limited diagnostic testing was performed pre-mortem and renal function, urinary analysis or 24-hour urine collections to assess proteinuria were not available.

In conclusion, the majority of urinary LAM-positive patients in our study had renal TB infection. Other mechanisms may contribute to LAM-antigenuria, however renal histological assessment did not reveal a prominent role for glomerular or tubular abnormalities. Moreover, urinary LAM LFA was found to be very sensitive and highly specific in the diagnosis of disseminated TB infection in hospitalized HIV-infected patients of whom 53% were not on anti-TB treatment at the time of death.
